# Conflicting interests: when whistleblowers profit from allegations of scientific misconduct

**DOI:** 10.1172/JCI166176

**Published:** 2022-11-01

**Authors:** Elizabeth M. McNally

The journal *Science* recently described a series of articles with potential scientific misconduct (1), emphasizing that many of them are connected to the developers of simufilam, an agent being evaluated to treat Alzheimer’s disease. *Science* called out potential image manipulation in multiple articles, including one published in the *Journal of Clinical Investigation* in 2012 (2). The *Science* story relayed many of the same details found in an April 18, 2022, *New York Times* piece (3).

The articles in *Science* and the *New York Times* focused primarily on the very serious topic of potential scientific misconduct. However, these articles only lightly touched upon the concept of short selling stock, and I believe this matter deserves more attention for its inherent conflicts of interest. Short selling entails borrowing shares of a stock, selling shares high, followed by buying shares back at a lower price and pocketing the difference. Short sellers profit from devaluing a stock. It is legal to devalue stocks using public statements, and short sellers will often use social media as a primary venue to make defamatory statements, expressed as opinions. This process is known as “short and distort.”

In August 2021, the Journal was contacted by email about the 2012 *JCI* article. The email asserted image manipulation, stating that sender was “retained by a law firm to investigate a concern of scientific misconduct related to the development of a drug intended to treat Alzheimer’s disease.” In the summer of 2021, the stock price connected to the company testing simufilam soared, drawing the interest of short sellers. This company developing simufilam is featured among the most highly shorted stocks (4), and by some estimates, over $100 million has been made from short selling this single stock. According to a profile in the *New Yorker*, two physician-scientists in 2021 worked with an attorney to petition the FDA to halt the clinical trials of simufilam (5). The FDA declined to consider the petition, but the filing itself was enough to send the stock price plummeting, garnering monetary gain for those who shorted the stock, as reported in the *New Yorker* and described in *Compliance Weekly* (5, 6).

Throughout 2022, the Journal has been repeatedly contacted to comment on the 2012 *JCI* paper. Although we cannot be certain, there now appear to be new “short and distorters.” A recent round of emails was sent simultaneously to multiple journals and editors, identifying 25 articles with potential problems and providing recommendations on how the journals should respond. Importantly, these accusatory emails do not identify any financial conflicts of interest on the part of the whistleblowers. The emails insist that an investigation begin within 24 hours and request that the journals update them on investigative progress. As an editor, I am expressing concern because this represents a new means of manipulating the scientific publishing industry.

The *JCI* has been at the forefront in trying to detect image manipulation in its manuscripts. For more than a decade, the *JCI* has required full uncut gel and blot images as part of the submission process. Beginning in 2021, the *JCI* was among the first to use an artificial intelligence–driven software package to analyze all images prior to accepting a paper (7, 8). Fundamentally, journals must trust that investigators are truthful about what is represented in their figures. Journals do not have access to primary data; journals have images that represent data. The primary data from experiments are held at the institutions where research is conducted. Soon, there will be more requirements to deposit primary data of publicly funded research in the public domain, and this may help journals improve in detecting scientific misconduct or even simple error. In all cases where findings erode our confidence in manuscripts published in the *JCI*, we will act accordingly through expressions of concern or retractions. There are ongoing institutional investigations for some of the allegations outlined in *Science*, and as a journal we await the outcome.

What is a journal to do? The *JCI* will always take seriously any allegations of misconduct or misrepresentation, but we will take the time needed to conduct a proper and thorough investigation. Going forward, whistleblowers, just like authors, editors, and referees, will be asked to inform us of recent, ongoing, and potential conflicts of interest. Financial conflicts of interest will be considered and weighed in any follow-up investigative actions and especially in any communication to the whistleblowers. We may independently seek to verify whistleblowers’ potential conflicts. We will limit what information we share with whistleblowers, since advanced notice of news reports positions short sellers to take advantage of shifts in stock prices. There is a time sensitivity to short selling, so we feel the best approach for any journal is to be deliberate and cautious, and to exert due diligence in investigating any allegations of scientific misconduct or data/image manipulation.

Last, if the Journal uncovers allegations made for the purposes of stock manipulation, with evidence of misinformation, the *JCI* may elect to express its concern to the US Securities and Exchange Commission or the Department of Justice.

## Version 1 11/01/2022

Electronic publication

## References

[1] Piller C (2022). Blots on a field?. Science.

[2] Talbot K (2012). Demonstrated brain insulin resistance in Alzheimer’s disease patients is associated with IGF-1 resistance, IRS-1 dysregulation, and cognitive decline. J Clin Invest.

[3] https://www.nytimes.com/2022/04/18/health/alzheimers-cassava-simufilam.html.

[4] https://finance.yahoo.com/screener/predefined/most_shorted_stocks/.

[5] https://www.newyorker.com/magazine/2022/01/24/jordan-thomas-army-of-whistle-blowers.

[6] https://www.complianceweek.com/risk-management/the-cassava-sciences-saga-short-sellers-gaming-the-fda-and-the-damaging-ripple-effects/31416.article#.

[7] Van Noorden R (2022). Journals adopt AI to spot duplicated images in manuscripts. Nature.

[8] Jackson S (2022). Data we can trust. J Clin Invest.

